# Genetically Modified (GM) Crop Use 1996–2020: Environmental Impacts Associated with Pesticide Use Change

**DOI:** 10.1080/21645698.2022.2118497

**Published:** 2022-10-13

**Authors:** Graham Brookes

**Affiliations:** PG Economics, Dorchester, UK

**Keywords:** Active ingredient, environmental impact quotient, GM crop, herbicide, insecticide, weed resistance

## Abstract

This paper assesses the environmental impacts associated with changes in pesticide use with GM crops at a global level. The main technologies impacting on pesticide use have been crops modified to be tolerant to specific herbicides so as to facilitate improved weed control and crops resistant to a range of crop insect pests that otherwise damage crops or typically require the application of insecticides to control them. Over the 24 year period examined to 2020, the widespread use of GM insect resistant and herbicide tolerant seed technology has reduced pesticide application by 748.6 million kg (−7.2%) of active ingredient and, as a result, decreased the environmental impact associated with insecticide and herbicide use on these crops (as measured by the indicator, the Environmental Impact Quotient (EIQ)) by a larger 17.3% between 1996 and 2020. The technology that has delivered the largest change in pesticide use has been insect resistant cotton, where a 339 million kg of active ingredient saving has occurred and the associated environmental impact (as measured by the EIQ indicator) has fallen by about a third.

## Introduction

Although the first commercial GM crops were planted in 1994 (tomatoes), 1996 was the first year in which a significant area of crops containing GM traits were planted (1.66 million hectares). Since then there has been a dramatic increase in plantings and by 2020, the global planted area was 185.6 million hectares (ha). In terms of the share of the main crops in which GM traits have been commercialized (soybeans, maize/corn, cotton, and canola), GM traits accounted for 47.4% of the global plantings to these four crops in 2020.

In addition, small areas of GM sugar beet (adopted in the USA and Canada since 2008), papaya (in the USA since 1999 and China since 2008), alfalfa (in the USA initially in 2005–2007 and then from 2011), squash (in the USA since 2004), apples (in the USA since 2016), potatoes (in the USA since 2015), and brinjal (in Bangladesh since 2015) have been planted.

There are two main categories of GM crop traits widely used that provide:

- Tolerance to the application of specific herbicides. The most commonly developed trait has been tolerance to glyphosate, followed by glufosinate in maize, cotton, canola (spring oilseed rape), soybean, sugar beet, and alfalfa. Since 2016, crops with additional tolerance to active ingredients like 2,4-D and dicamba have been introduced, mostly in North America. This GM Herbicide Tolerant (GM HT) technology allows for the ‘over the top’ spraying of the (GM HT) crops with these specific herbicides, that target both grass and broad-leaved weeds but do not harm the crops themselves;
- Resistance to specific insect pests of maize, cotton, soybeans, and brinjal. This GM insect resistance (GM IR), or ‘Bt’ technology offers farmers resistance in the plants to major pests, such as stem and stalk borers, earworms, cutworms, and rootworm (eg, *Ostrinia nubilalis, Ostrinia furnacalis, Spodoptera frugiperda, Diatraea spp, Helicoverpa zea, and Diabrotica spp*) in maize, bollworm/budworm (*Heliothis sp and Helicoverpa*) in cotton, caterpillars (*Helicoverpa armigeru)* in soybeans and the fruit and shoot borer (*Leucinodes orbanalis)* in brinjal. Instead of applying insecticide for pest control, a very specific and safe insecticide is delivered via the plant itself through ‘Bt’ gene expression.

In addition, the GM papaya and squash referred to above are resistant to important viruses (eg, ringspot in papaya), the GM apples are non-browning and the GM potatoes (first planted in 2016) have low asparagine (low acrylamide which is a potential carcinogen) and reduced bruising.

This paper presents estimates of some of the main environmental impacts associated with the widespread use of crops containing these GM traits by focusing on changes in the use of insecticides and herbicides applied to the GM crops relative to conventionally grown alternatives.

The study integrates data for 2019 and 2020 into the context of earlier developments and updates the findings of earlier analysis presented by Brookes and Barfoot, 2020.^1^

The aim has been to provide an up-to-date and as accurate as possible assessment of some of the key environmental impacts associated with the global use of GM crops. It is also hoped the analysis continues to make a positive contribution to better understanding of the impact of this seed technology and facilitates more informed decision-making, especially in countries where the planting of crops containing GM technology is currently not permitted.

## Methodology

This analysis draws on a combination of existing literature and analysis by the author of crop and country-specific farm-level changes in husbandry, pest and weed control practices, and pesticide (herbicide and insecticide) usage data. In particular, the analysis of changes to herbicide usage and tillage practices with GM crops takes into consideration how farmers have made changes to weed control practices so as to address weed resistance development to the main herbicide (glyphosate) used with GM HT crops.

### Impact Associated with Use of Insecticides and Herbicides

The control of pests and reduction of weed competition in agricultural production systems is vital if adequate quantities of good-quality food are to be consistently made available to feed the (growing) world’s population. The primary way in which this has been delivered in global agriculture has been through the use of pesticides, which have therefore made important contributions to improving crop yields, the quality of produce and in turn, improved global food security (Cooper and Dobson, 2007,^2^ Aktar, Sengupta and Chowdhury, 2009.^3^) However, despite these benefits, pesticides can be hazardous to humans and the environment, having the potential to disperse into the environment and contaminate/poison non-target species. The consequences of pesticide use have been documented and discussed by many (eg, Bourget and Guillemaud, 2016,^4^ Sud, 2020,^5^) with pesticide regulatory systems in place ‘*to balance the societal and economic benefits with the unintentional and undesirable environmental and health impacts’* (Lewis et al, 2021.^6^)

Against this background, the regulatory approval and subsequent widespread commercial availability of GM crops in the mid-1990s ushered in a major change to how some pests and weeds were controlled in the crops where the seed technology has become widely used (canola, cotton, maize and soybeans).

An important component of any assessment of the environmental impact of widespread GM crop cultivation is therefore identifying how their use has impacted on pest and weed control practices and, in particular, on how insecticide and herbicide use has altered. In turn, this requires comparisons of the respective weed and pest control measures used on GM versus the ‘conventional alternative’ form of production and how the use of pesticides in each production system may disperse into the environment and have negative consequences for non-target species. This presents a number of challenges.

#### How to Measure Environmental and Human Health Impacts: An Appropriate Indicator

The challenge to developing an appropriate indicator for evaluating potential non-target effects of pesticide use requires taking account of differences in terms of environmental and human health impacts of the numerous pesticide active ingredients used and be sensitive enough to take account of differences to the amount (of each) applied to crops. An indicator also needs to adaptable enough to take account of changes in the availability of pesticides via the withdrawal of some from use and the introduction of new chemistry to the marketplace.

To assess the risks and impact of pesticide use in a consistent but simplified manner a number of indicators have been developed. Some of these are hazard-based, whilst others try to consider risk.

The most common (and crudest) form of indicator used (and which has been most commonly been presented in the literature relating to the impact associated with GM crop use) has been simply to examine impact in terms of the volume (quantity) of pesticide active ingredient applied under the principle that ‘more is bad for the environment and less is better for the environment.’ Whilst this approach is sensitive enough to take account of changes in the amount applied and can accommodate the changing availability of different active ingredients, it is not a good measure of environmental impact because the toxicity of each pesticide is not directly related to the amount (weight) applied and there is no consideration of how the active ingredients disperse into the environmental or may affect non-target species.

There exist alternative measures that could be used. These include:

- The Environmental Impact Quotient (EIQ) developed at Cornell University by Kovach et al. 1992^7^ and updated annually. This hazard-based indicator is one of the earliest indicators developed and uses a scoring system approach to determine the impact of a pesticide on humans, groundwater, and bio-diversity. Rating values are given for effects on farm workers, consumers, toxicity to beneficial insects, toxicity to bees, fish and birds, plant surface half-life, chronic health effects, run-off and leaching potential, soil residue half-life, and mode of action. These are added to produce a single EIQ rating per active ingredient, with the EIQ indicator value for each active ingredient determined by the amount applied;
- The Environmental Yardstick for Pesticides developed in the Netherlands, which quantifies risk of pesticide use at the field, regional and national level by allocating environmental impact points for risks to groundwater, aquatic species, and soil organisms (Reus and Leenderste, 2000^8^);
- SYNOPS indicator, developed to support the German National Action Plan on the Sustainable Use of Plant Protection Products (Strassemeyer and Gutsche, 2010^9^) which assess the acute and chronic pesticide risks to soil and surface water and non-target pollinator species through the calculation of Predicted Environmental Concentrations (PECs);
- The Norwegian pesticide risk indicator (NERI) was developed both as a tool to assess pesticide use risk and as a method for taxation of pesticides (Stenrød et al., 2008.^10^) It uses a rating system for human health impacts (four risk classes according to the risk phrases on product labels) and environmental risk by adding up rating scores for effects on earthworms, bees, birds, aquatic organisms, mobility and leaching potential, persistence, bioaccumulation and formulation type. The accumulated rating scores are then used to classify pesticides into three environmental risk classes and grouped into several classes subject to different tax levels;
- Like NERI, the Pesticide Load (PL) risk indicator developed in Denmark was primarily developed to form the basis for a national pesticide tax system (Kudsk et al., 2018.^11^) The indicator is made up of three categories of indicator that aim to measure the potential pressure on human health, environmental fate, and ecotoxicity. This does not measure actual impact but aims to reflect the relative environmental pressures that arise from the differing hazards of each pesticide and the amount of each applied. In relation to the environmental fate ecotoxicity indicators, pesticide loading points for each pesticide is measured against a benchmark reference substance that is classified as the most harmful active ingredient for each parameter (eg, longest soil half-life) and all other substances are expressed relative to this reference active ingredient;
- Total Applied Toxicity (TAT). This indicator uses regulatory threshold levels for impacts of pesticide active ingredients on eight different groups of non-target species as indicators of potential impact on bio-diversity (Schulz et al., 2021.^12^) The authors applied this approach to assess environmental impacts associated with insecticide and herbicide use in maize and soybeans in the USA;
- The Ecological Relative Risk (EcoRR) indicator (Sanchez-Bayo et al., 2002.^13^) This is a site-specific indicator that assesses and compares the ecotoxicological risk of pesticides on ecosystems. It compares relative risks between different pesticides and assesses the potential ecological impact of their residues. The EcoRR approach is based on standard frameworks for risk assessment (eg, Predicted Environmental Concentration (PEC) or toxicity) but takes account of factors, such as persistence of residues and biodiversity of ecosystems;
- The European Union’s Harmonized Environmental Indicators for Pesticide Risk (HAIR^14^) is a set of indicators developed for calculating trends in aggregated risks associated with the agricultural use of pesticides. This is a series of models for the evaluation of the environmental fate of pesticides (eg, PEARL – Pesticide Emission Assessment at Regional and Local scales that model’s pesticide behavior in the soil-plant system.^15^) It classifies pesticides used in European agriculture into different risk categories so as to set a baseline for the reduction in the aggregate level of risks associated with pesticide use. Success (or failure) in this goal is largely determined via monitoring and collecting data on the sales of products in each pesticide risk category, with sales figures used as a proxy for use (and ultimate impact).

Whilst the list of indicators above represent some, not a full list of those available, it highlights the range of indicators, each of which was originally developed with a specific purpose in mind (eg, as a vehicle for establishing a pesticide tax) and/or targeting specific local, regional, or national impact assessment. Hazard-based indicators do not assess risk or probability of exposure to pesticides typically rely to some extent on qualitative assumptions and ratings drawn from product label and regulatory threshold information for the scaling and weighting of (quantitative) risk information. This can result, for example, in the case of the EIQ indicator, in a low-risk rating for one factor (eg, impact on farm workers) possibly canceling out a high-risk rating factor for another factor (eg, impact on ecology). Other models or indicators that attempt to include risk of exposure into the assessments typically require site-specific or local/regional-level data on issues such ground water levels or soil structure or at least the application of standard scenario models for exposure at a number of locations. This is why indicator/models such as SYNOPS, which was developed for specific applicability to Germany, are country-specific in their application and replication/adaption of such models to other countries (as is the case in respect of Norway and Switzerland) is difficult, time-consuming and requires adaption to reflect differing levels of information.

For the purposes of our analysis, an indicator is required that is readily available and applicable across a range of crops (notably the four main crops where GM crop technology is widely used), grown in more than 20 countries on an area of approximately 186 million hectares (2020). This encompasses a wide range of climates, soil types, weather, agricultural production systems, ecosystems, non-target species, and pest and weed control practices. Whilst a risk rating type model, such as SYNOPS might represent an ideal to utilize, the transferability/applicability of such an exercise to this context is undeliverable.

The indicator that has been used most by various analysts to assess the environmental impact associated with changes to pest and weed control practices with the growing of GM crops has been the EIQ. It was originally developed to allow for the comparison of the environmental impact of different crop protection systems used as more integrated forms of pest and weed control were introduced in the USA, in the 1990s. Its early adaption and use in respect of the use of GM crop technology relative to conventional (non-GM) cropping was made by Brimner et al., 2004^16^ and Kleiter, 2005,^17^ with this author also using it first in 2005 (Brookes, 2005.^18^) It has also subsequently been used by others (eg, Biden et al., 2018.^19^) As indicated above, the EIQ integrates the various rating values for effects on farm workers, consumers, toxicity to beneficial insects, toxicity to bees, fish and birds, plant surface half-life, chronic health effects, run off and leaching potential, soil residue half-life and mode of action to produce a single EIQ rating per active ingredient, with the EIQ indicator value (or field EIQ) for each active ingredient determined by the amount applied. For example, the EIQ rating for glyphosate is 15.33. By using this rating multiplied by the amount of glyphosate used per hectare (eg, a hypothetical example of 1.1 kg applied per ha), the field EIQ value for glyphosate would be equivalent to 16.86/ha. The EIQ indicator used is therefore a comparison of the sum of the field EIQ/ha for each pesticide used for a conventional (non-GM) versus GM crop production system, with the total environmental impact or load of each system, a direct function of respective field EIQ/ha values and the area planted to each type of production (GM versus conventional).

The author of this analysis have used the EIQ indicator now for several years because it:

- Summarizes significant amounts of information on pesticide impact into a single value that, with data on usage rates (amount of active used per hectare) is transferable and relatively easy to use/apply across crops and production systems across the many diverse regions and countries where GM crops have been widely grown, especially as EIQ values have been computed for about 180 herbicide active ingredients and 145 insecticide active ingredients;
- Provides an improved assessment of the impact of GM crops on the environment when compared to only examining changes in volume of active ingredient applied, because it draws on some of the key toxicity and environmental exposure data related to individual products, as applicable to impacts on farm workers, consumers, and ecology.

Whilst utilizing the EIQ indicator in this analysis, the author acknowledges that it is only a hazard indicator and, as indicated above, it has important weaknesses. These have been discussed by others, such as Peterson R and Schleier J 2014^20^ and Kniss A and Coburn C 2015.^21^ In an ideal world, this assessment would use a risk-based and more comprehensive indicator (eg, SYNOPS). However, undertaking such an exercise at a global level would require a substantial and ongoing input of labor, time, and site-specific data. It is therefore not surprising that no such exercise has, to date been undertaken. It is hoped that in the near future indicators that better consider risks of pesticide exposure and the fate of pesticides in the environment but at the same time are transferable and reasonably easy to use and apply to a variety of different cropping systems around the world will become available for undertaking an analysis of this nature.

Despite the acknowledged weaknesses of the EIQ as an indicator of environmental impact associated with pesticide use, the author of this paper continues to use it because no other indicator currently offers the scope for relatively easy transferability and use across a wide range of crops, and countries. In this paper, the EIQ indicator is used in conjunction with examining changes in the amount of pesticide active ingredient applied to GM crops relative to conventional (non-GM) crops.

#### Availability and Representativeness of Data

Assessing the environmental impact associated with crop protection and weed control practices used with GM crops relative to conventional alternatives, requires making comparisons of the methods used, most notably relating to the use of insecticides and herbicides. Such comparison data ideally derives from farm-level surveys that collect usage data on the different forms of production. A search of the literature on insecticide or herbicide use change with GM crops shows that the number of studies exploring these issues is fairly limited (eg, Qaim and Traxler, 2005,^22^ Pray C, 2002^23^) with even fewer (eg, Brookes, 2005,^18^ Brookes, 2008^24^) especially in terms of providing data to the individual active ingredient level. Secondly, national-level pesticide usage survey data is also limited. There are no published, detailed, annual pesticide usage surveys conducted by national authorities in any of the countries currently growing GM crop traits. Of the GM crop growing countries, the USA, through the US Department of Agriculture (USDA) is the only country that regularly publishes pesticide usage surveys on some of the crops in which GM technology is used. However, these are not conducted on an annual basis for each crop (eg, the last time maize was included was 2018 and previous to this, in 2016, 2014, 2010, and 2005, for soybeans the last time included was 2020 and before that 2018 and 2015) and do not disaggregate usage by production type (GM versus conventional).

The only sources of pesticide usage data on the crops in which GM technology has been used around the world derive from two main sources:

- Ad hoc/bespoke studies of the impact of using GM crop technology relative to conventional alternative crops. These are typically crop specific and limited both in terms of time periods covered (1–3 years) and may be local in nature rather than reasonably representative of a national perspective;
- Private market research companies that undertake farm-level surveys of crop-specific pesticide use on a regular basis. These are primarily conducted to collect data (typically to the product and brand level) in order to service the market intelligence and information requirements of businesses that sell crop protection products and can be found in many countries. The most comprehensive datasets from these sources typically focus on usage in the larger agricultural producing countries such as the US, Canada, Brazil, Argentina, Paraguay, South Africa, the EU, Australia, China, and India. Access to these datasets requires the payment of subscriptions to the suppliers and typically comes with restrictions on the publication of disaggregated levels of data (in particular to the brand and product level). With the exception of the data available relating to pesticide use on a limited range of crops in the USA, these sources of data do not differentiate between use of active ingredients or products by production type (GM versus conventional).

In this analysis, the author draws on both categories of data. This includes reviewing literature on published GM crop impact studies both in peer reviewed and other literature. It draws on relevant publicly available pesticide usage survey data (primarily USDA) and has accessed private market research sources from the primary providers over the last 20 years, most notably Kynetec and Kleffmann, which have both collected pesticide usage data on many crops in a number of countries around the world. Details of the sources used for analysis by crop and country are detailed in Appendix 3. This means that the analysis draws on the most comprehensive and detailed sources of pesticide usage available at a global level. It also means that the EIQ indicator calculations take into consideration all of the main pesticides used in the production of the crops where GM crop technology is used. For example, in the USA, this relates to about 55, 50, and 40 herbicides, respectively, used in soybeans, maize, and cotton crops, in Brazil, 40 and 30 herbicides, respectively, used on soybean and maize crops and in Argentina, about 60 and 20 herbicides used on soybeans and maize. Similarly, EIQ values associated with the use of about 30 insecticides used in cotton grown in Australia and India, 45 in China, and about 50 in the USA.

Whilst the research has accessed and utilized these comprehensive sources of (pesticide use) data, it is important to recognize the limitations that come with this data for the purposes of the analysis.

A primary objective of the research is to assess the environmental impact of crop protection practice differences between production systems that use GM crop technology and those that use conventional technology (based on the EIQ indicator). In order to do this herbicide and insecticide usage changes with GM crop technology adoption require identification in terms of not only what is currently used with GM crops, but also in the ‘counterfactual situation,’ that is, what herbicides/insecticides might reasonably be expected to be used in the absence of crop biotechnology on the relevant crops (ie, if the entire crops reverted to using non-GM production methods).

The most straightforward way of doing this is from observations and surveys of crop protection practices on farms using the different production systems. As such, this source of data from ad hoc/bespoke surveys of usage on farms using GM versus non GM technology has been utilized in this analysis, where available. This category of data does, however, not cover every trait, crop, country, or year, and therefore alternative sources and assumptions are required.

As indicated above, an important source of data used is the regular farm-level pesticide usage survey data collected by private market research companies and made available on subscription. For all countries studied, with the exception of the USA, this data source does not disaggregate usage for each active ingredient to production type (GM versus non-GM) and therefore this source has been used in the following way:

- Where GM-herbicide traited crops account for all or almost all (90% plus) of production in a country (eg, soybeans in all of the South American countries), the herbicide (and/or insecticide) usage data is assumed to represent the GM HT and/or GM IR crop;
- For conventional crops and GM HT/IR crops where the less than 90% of the crop is GM HT/IR, estimates of herbicide use in overall weed/pest control systems are based on information drawn from extension and industry advisors in each country (some of this derives from literature such as weed control guides from extension services and some from direct contact with advisors in such services, academics, and industry representatives). In all cases, the aim has been to identify the weed/pest control practices that farmers might reasonably be expected to use (including typical herbicide and insecticide application rates) in both GM HT/IR and conventional crops. In relation to GM IR versus conventional crop pest control practices, the focus has been on products used to control only the pests that the GM IR technology targets control (typically lepidopteran pests (and rootworm in North America)) and not products used to control other categories of pests (eg, sucking pests and (cotton) weevils). In addition, in the early years of adoption of GM traited crops, the usage assumed for conventional crops has been cross-checked with recorded usage levels in the years immediately prior to the introduction of GM technology to ensure that usage levels derived from the extension service approach did not over (or understate) likely usage levels.

In the case of the US, where pesticide usage data for the crops of cotton, maize, and soybeans is available at a disaggregated level (GM versus conventional crop), the approach used reflects the relative balance of the total crop accounted for GM versus conventional as follows:

- Recorded herbicide and insecticide usage (to the active ingredient level) for GM traited crops has been used for all years;
- Recorded herbicide and insecticide usage (to the active ingredient level) for conventional crops has been used for all years until the conventional share of total production fell below 30% of the crop (2001 for cotton and soybeans and 2007 for maize: statistical source: USDA NASS 2022);
- For conventional cotton and soybeans post 2001 and maize post 2007, estimates based on extension service-type sources have been used.

In the case of the USA analysis, the reasons why herbicide/insecticide usage levels identified for the increasingly small conventional crop were not used and have been replaced by usage patterns identified from ‘extension service’ type sources reflects the author’s assessment that these levels of usage are unrepresentative of what might reasonably be expected if all of the (majority) area using GM technology reverted to conventional (non-GM) production systems. More specifically:

- Although pest/weed damage and competition varies by year, region and within region, farmers’ who consistently farm conventionally rather than using GM seed may be those with relatively low levels of pest/weed problems, and hence see little, if any, economic benefit from using the GM traits targeted at these pest/weed problems. In addition, late or non-adopters of new technology in agriculture are typically those who generally make less use of newer technologies than earlier adopters. As a result, insecticide/herbicide usage levels on non-adopting (conventional) farms tend to be below the levels that would reasonably be expected on an average farm with more typical pest/weed problems and where farmers are more willing to adopt new technology (see example below);

- Some of the farms continuing to sow conventional seed, use extensive, low-intensity production methods (including organic) which feature, limited (below average) use of herbicides/insecticides. If the pesticide usage patterns of this sub-set of growers are used as a proxy to represent usage patterns if all farmers returned to farming without GM technology, this is likely to understate pesticide usage for the majority of farmers. For example, prior to the adoption of GM HT cotton in the USA in the mid-1990s, when all of the crop was conventional, about 90% of the crop was typically receiving some use of herbicides for weed control and the average amount of herbicide used (kg/ha) and average EIQ/ha value for herbicide use on this crop were, respectively, about 2.54 kg ai/ha with 54/ha. Within this cotton farmers in Texas, where many producers practice extensive production systems (little or no use of inputs like pesticides), the average amount of herbicide active ingredient used and average EIQ/ha were respectively about 1.54 kg/ha and an average EIQ/ha of 31.1 (Texas then accounted for just under 60% of the USA cotton crop). In 2020, the conventional cotton crop accounted for about 4% of the total crop (about 138,000 ha), of which 84% of the crop received some form of herbicide use as part of weed control and 80% of the crop was located in Texas. Using the recorded average usage figures for herbicide use in Texas in 2020 (about 1 kg ai/ha and an EIQ/ha value of about 21/ha) as a proxy for what might be used if all of the USA cotton reverted to conventional (non-GM) production methods, is therefore likely to significantly understate usage;

- The widespread adoption of GM IR technology has resulted in ‘area-wide’ suppression of target pests in maize, cotton, and soybean crops. As a result, conventional farmers (eg, of maize in the USA) have benefited from this lower level of pest infestation and the associated reduced need to apply insecticides (Hutchison et al., 2010.^25^)

- Many farmers have experienced improvements in pest/weed control with GM technology compared to the conventional control methods previously used. If these farmers were to switch back to using conventional techniques, it is likely that most would want to maintain pest/weed control levels obtained with GM traits and therefore some would probably use higher levels of insecticide/herbicide than they did in the pre-GM crop days (eg, as identified by Brookes ^200824^ relating to IR maize growers in Spain). Nevertheless, the decision to use more pesticide or not would be made according to individual assessment of, for example, the potential benefits (eg, from higher yields) compared to the cost of additional pesticide use.

This methodology has been used by others in relation to analysis of pesticide use change with GM crops in the US, such as Sankala and Blumenthal, 2003,^26^ Sankala and Blumenthal, 2006^27^ and Johnson and Strom, 2006.^28^

Details of how this methodology has been applied to the 2020 calculations, sources used for each trait/country combination examined and examples of typical conventional versus GM pesticide applications are provided in Appendices 1 and 2. Data sources used in the analysis are shown in Appendix 3.

## Results and Discussion

### HT Crops

**Figure 1.**
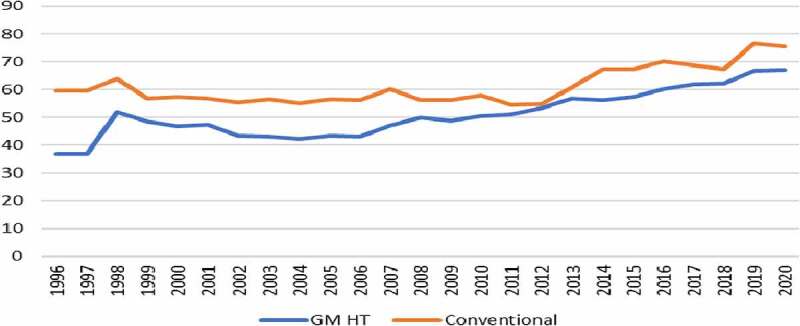
A comparison of the average EIQ per ha value for weed control systems used in conventional maize that delivers equal efficacy to weed control systems in GM HT maize in the USA 1997–2020.

Sources: Sankala & Blumenthal 2006,^27^ Johnson & Strom 2008^28^ and updated for this research for 2009–2020, based on University Extension Services, Industry, USDA NASS, and Kynetec

One of the most striking impacts associated with use of GM HT (largely tolerant to glyphosate) seed technology has been how the nature and profile of herbicides used has changed. Before the availability of GM HT technology, weed control in most crops was based on the use of a fairly broad range of, mostly selective (grass weed and broad-leaved weed) herbicides. With widespread availability of GM HT seed technology, this practice was largely replaced by use of one or two broad-spectrum herbicides (mostly glyphosate) used in conjunction with a small number of other (complementary) herbicides (eg, 2,4-D). This resulted in:

- Aggregate reductions in both the volume of herbicides used (in terms of weight of active ingredient applied) and the associated field EIQ values when compared to usage on conventional (non-GM) crops in some countries, indicating net improvements to the environment. Specific crop/country combinations where this has occurred include GM HT soybeans and maize in the USA, Canada, and South Africa (see crop/trait-specific discussion below and a detailed example for GM HT soybeans in Canada in Appendix 1);
- In other countries, the switch to using GM HT technology resulted in a net increase in the amount of herbicide active ingredient used when compared to usage on the conventional crop alternative. However, in terms of the associated environmental impact, as measured by the EIQ indicator, the environmental profile of the GM HT crop has usually been better than its conventional equivalent. See, for example, GM HT soybean usage in South American countries presented below, together with a detailed example for GM HT soybeans in Argentina in Appendix 1;
- After a number of years of widespread use of GM HT (tolerant to glyphosate) crop technology, incidences of weed resistance to glyphosate began to increase (see more detailed discussion below) and became a major problem in some regions (see www.weedscience.org). This can be attributed to how glyphosate was originally used with GM HT crops. Due to glyphosate’s highly effective, broad-spectrum post-emergence activity, it was often used as the sole method of weed control by many early users of GM HT crops. This put tremendous selection pressure on weeds and contributed to the evolution of weed species populations where individual plants were increasingly resistant to control with glyphosate alone. In addition, the facilitating role of GM HT technology (CTIC, 2002,^29^ ASA, 2001^30^) in the adoption of NT and RT production techniques in North and South America contributed to the emergence of weeds resistant to herbicides like glyphosate and to weed shifts toward those weed species that are not inherently well controlled by glyphosate. As a result, since the early 2000s, growers of GM HT crops have been increasingly advised to adopt more integrated weed control practices, which use other herbicides (with different and complementary modes of action) in combination with glyphosate and in some cases to adopt cultural practices (eg, revert to plowing: Vencill et al., 2012,^31^ Norsworthy et al., 2012.^32^) Also, in the last 5 years, GM HT crops tolerant to additional herbicides (usually providing multiple herbicide tolerances in a crop) such as 2,4-D, dicamba and glufosinate have become available and are widely grown in North America in 2020. At the macro level, these changes have influenced the mix, total amount, cost and overall profile of herbicides applied to GM HT crops. This means that compared to the early 2000s, the amount and number of herbicide active ingredient used with GM HT crops in most regions has increased, and the associated environmental profile, as measured by the EIQ indicator, deteriorated (see Fig. 1). This increase in the amount of herbicide used has been cited by some (eg, Benbrook, 2012^33^) as an environmental failing of the technology. However, this fails to acknowledge that a similar trend in herbicide use and associated environmental profile (higher level of active ingredient use and a deteriorating EIQ profile) has occurred in conventional crops in order to address resistance issues in weeds to non-glyphosate herbicides that are more commonly used in conventional crops than GM HT (tolerant to glyphosate) crops. This is discussed further below in the more detailed analysis of weed resistance issues. Thus, despite this trend, in 2020, the environmental profile of GM HT crop use, as measured by the EIQ indicator, has continued to represent an improvement compared to the conventional alternative (see for example Fig. 1). It is also worthy of note that many of the herbicides used in conventional production systems had significant resistance issues themselves in the mid-1990s and this was one of the reasons why glyphosate tolerant soybean technology was rapidly adopted, as glyphosate provided good control of these resistant weeds (for example, the Weedscience.org database of herbicide-resistant weeds show that there were approximately 50 weeds commonly found in soybean crops that were exhibited resistance to some herbicides used at that time).

These points are further illustrated in the analysis below which examines changes in herbicide use by crop over the period 1996–2020 and specifically for the latest year examined, 2020.

### GM HT Soybean

**Figure 2.**
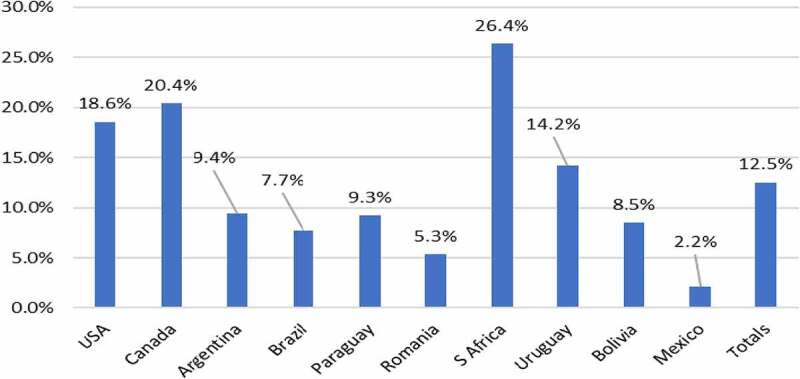
Aggregate EIQ changes (improvements) with use of GM HT soybeans: by adopting country 1996–2020.

Note: see appendix 1 for details of how these changes are calculated relative to the conventional (non-GM) baseline

The environmental impact of herbicide use change associated with GM HT soybean adoption between 1996 and 2020 is summarized in Table 1 and Fig. 2. Overall, there has been a small net decrease in the amount of herbicide active ingredient used (−0.1%) at the global level (countries using the technology), which equates to 3 million kg less active ingredient applied to these crops than would otherwise have occurred if a conventional crop had been planted. At the country level, there has been a net reduction in the amount of herbicide active ingredient used relative to the conventional alternative in the USA, Canada, and South Africa whilst in the other adopting countries there has been a net increase in usage (Table 1). In contrast, the environmental impact, as measured by the EIQ indicator, improved overall by 12.5%, with improved EIQ profiles in all user countries (Fig. 2).

**Table 1.** GM HT soybean: summary of active ingredient usage changes by adopting country 1996–2020.

Country	Change in active ingredient use (million kg)	%
Romania (to 2006 only)	+0.04	+4.3
Argentina	+7.67	+0.6
Brazil	+7.89	+0.5
USA	−26.5	−1.8
Canada	−3.0	−5.1
Paraguay	+8.5	+6.8
Uruguay	+0.7	+1.6
South Africa	−1.4	−9.9
Mexico	+0.03	+2.0
Bolivia	+3.0	+6.7
**Aggregate impact: all countries**	**−3.07**	**−0.1**

Notes: Negative sign = reduction in usage. Positive sign = increase in usage

In 2020, the amount of herbicide active ingredient applied to the global GM HT soybean crop decreased by a marginal 0.07 million kg (−0.02%) relative to the amount reasonably expected if this crop area had been planted to conventional soybeans. This highlights the point above relating to recent increases in herbicide use with GM HT crops to address weed resistance issues. Despite these increases in the volume of active ingredient used, in EIQ terms, the environmental impact of the 2020 GM HT soybean crop (at the global level in countries using the technology) continued to represent an improvement relative to the conventional alternative (a 9.3% improvement).

### GM HT Maize

**Figure 3.**
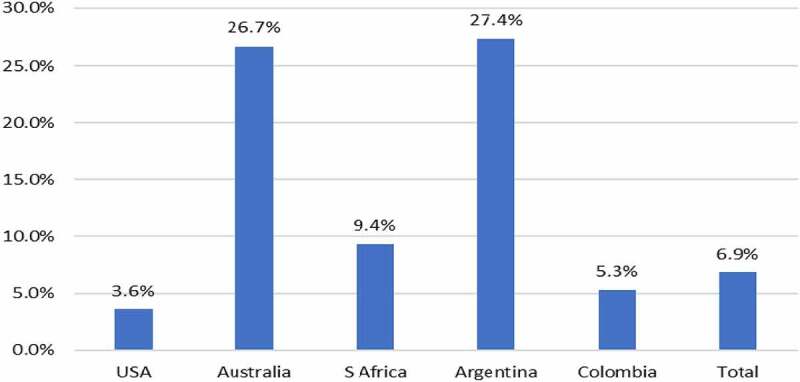
Aggregate EIQ changes (improvements) with use of GM HT maize: by adopting country 1997–2020.Note: see appendix 1 for details of how these changes are calculated relative to the conventional (non-GM) baseline

The widespread adoption of GM HT maize technology since 1997 has resulted in an aggregate reduction in the volume of herbicide active ingredient used on the global area of this crop of 224 million kg of active ingredient (−6.2%: Table 2). Net reductions in the volume of herbicide active ingredient usage relative to the conventional alternative were recorded in the USA, Canada, South Africa, Colombia, and Vietnam, with net increases in the volume used in South American user countries (and Philippines: Table 2). In terms of the associated environmental impact, as measured by the EIQ indicator, the aggregate change recorded was an improvement of 7.8%, with all user countries recording a net environmental improvement (Fig. 3).

**Table 2.** GM HT maize: summary of active ingredient usage 1997–2020: by adopting country.

Country	Change in active ingredient use (million kg)	%
USA	−220.3	−8.7
Canada	−6.6	−8.0
Argentina	+12.8	+5.5
South Africa	−1.4	−1.1
Brazil	−8.1	+1.4
Uruguay	+0.02	+6.1
Vietnam	−0.03	−2.2
Philippines	+0.1	+0.6
Colombia	−0.3	−5.1
**Aggregate impact: all countries**	**−223.8**	**−6.2**

Notes: 1. Negative sign = reduction in usage. Positive sign = increase in usage, 2. Paraguay not included due to lack of available data on herbicide use

**Figure:**
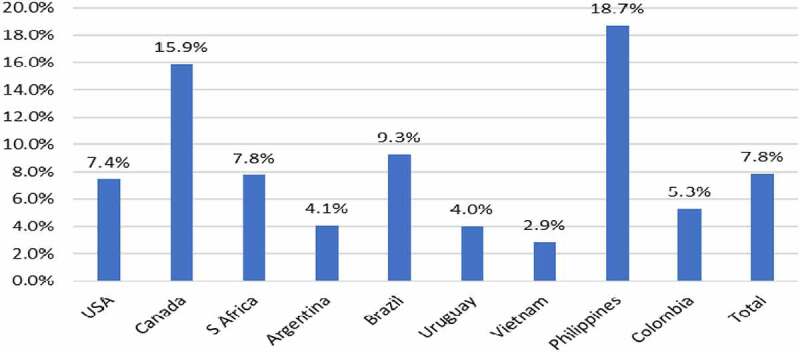

In 2020, there was also a net reduction in herbicide usage relative to the amount expected if this crop area had been planted to conventional maize equal to 5.1 million kg of active ingredient (−2.4%), with a larger aggregate environmental improvement, as measured by the EIQ indicator of 11.7%.

### GM HT Cotton

**Figure 4.**
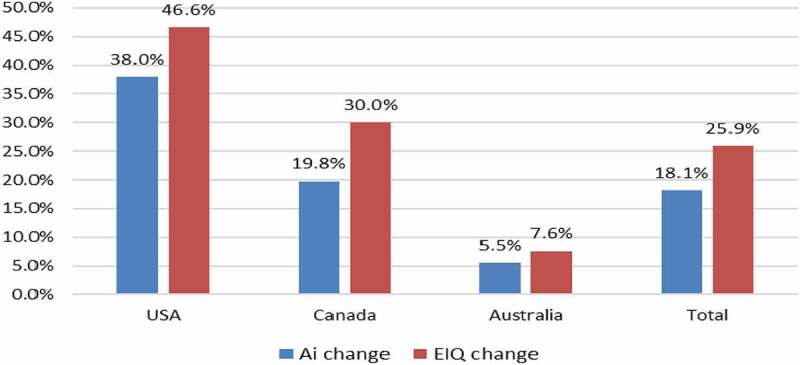
Aggregate herbicide active ingredient usage (reductions) and EIQ changes (improvements) with use of GM HT canola: by adopting country 1996–2020.

Note: see appendix 1 for details of how these changes are calculated relative to the conventional (non-GM) baseline

A similar pattern of change has occurred with the adoption of GM HT cotton since 1997. There has been a net aggregate reduction in herbicide active ingredient use of 38.6 million kg over the 1997–2020 period (−8.4%) with net reductions in the amount of herbicide active ingredient used relative to the conventional alternative in all adopting countries, except South Africa, where there has been a marginal increase in net usage (Table 3). This represents an 8.4% reduction in aggregate usage. In terms of the associated environmental impact, as measured by the EIQ indicator, there has been an aggregate net improvement of 6.9% net environmental improvement, with improvements in the environmental profile found in all adopting countries (Fig. 4). In 2020, the use of GM HT cotton technology resulted in a 0.6 million kg reduction in herbicide active ingredient use (−3%) relative to the amount expected if this crop area had been planted to conventional cotton. In terms of the associated environmental profile, as measured by the EIQ indicator, this represents an 8.7% environmental improvement.

**Table 3.** GM HT cotton summary of active ingredient usage 1997–2020: by adopting country.

Country	Change in active ingredient use (million kg)	%
USA	−25.7	−6.5
South Africa	+0.01	+0.5
Australia	−6.5	−20.5
Argentina	−6.1	−26.3
Colombia	−0.3	−5.1
**Aggregate impact: all countries**	**−38.6**	**−8.4**

Notes: 1. Negative sign = reduction in usage. Positive sign = increase in usage, 2. Other countries using GM HT cotton – Brazil and Mexico, not included due to lack of data

### Other HT Crops

GM HT canola (tolerant to glyphosate or glufosinate) technology was first grown in Canada in 1996. Since then the USA adopted the technology in 1999 and farmers in some states in Australia started to use it in 2008. The use of this technology has resulted in a significant reduction in the aggregate amount of herbicide active ingredient used relative to the amount expected if this crop area had been planted to conventional canola (−18.1%). The associated environmental impact, as measured by the EIQ indicator improved by a larger 25.9% (Fig. 4).

In 2020, the use of GM HT canola technology continues to facilitate a net aggregate reduction in the amount of herbicide active ingredient used equal to 2.3 million kg (−20.3%) relative to the amount reasonably expected if this crop area had been planted to conventional canola and an improvement in associated environmental impact, as measured by the EIQ indicator of 25.4%.

GM HT (tolerant to glyphosate) sugar beet has been grown in the USA and Canada since 2008. The adoption of this technology led to changes in weed control practices based on a combination of several applications of selective herbicides and some use of mechanical and hand weeding to a more herbicide – focused weed control regime based largely (but not exclusively) on glyphosate. As with the other GM HT crops adopted in North America, early weed control practices with GM HT sugar beet were dominated by the use of glyphosate and little or no use of additional herbicides and net reductions in the volume of herbicides used occurred. Then, as the incidence of weeds becoming resistant to glyphosate increased, the amount of herbicide used on GM HT sugar has increased, as farmers have applied other herbicides with different modes of action to glyphosate to control these weeds. As a result, over the period 2008–2020, the net aggregate impact of using GM HT technology in the USA and Canadian sugar beet crops has been a small net reduction in the total volume of herbicides applied to the sugar beet crop relative to the amount expected if this crop area had been planted to conventional sugar beet equal to about 0.8 million kg of active ingredient used (−4.4%). In terms of the associated environmental impact, as measured by the EIQ indicator, there has been a net 15.5% improvement.

In 2020, the use of GM HT technology in the sugar beet crops in these countries has resulted in a net increase of 69,200 kg of herbicide active ingredient usage (+4.4%) relative to the amount reasonably expected if this crop area had been planted to conventional sugar beet and, in terms of the net associated environmental impact, as measured by the EIQ indicator, this has been largely neutral.

### Weed Resistance

As indicated above, weed resistance to glyphosate has become a major issue affecting many farmers using GM HT (tolerant to glyphosate) crops. Worldwide there are currently (accessed August 2022) 56 weeds species resistant to glyphosate of which some are not associated with glyphosate tolerant crops (Heap I International Survey of Herbicide Resistant Weeds – www.weedscience.org). For example, this dataset shows that in the USA, there are currently 17 weeds recognized as exhibiting resistance to glyphosate, of which two are not associated with glyphosate tolerant crops. In addition, the first glyphosate-resistant weeds developed in Australia in the mid-1990s before the first adoption of GM HT crops (GM HT cotton in 2000) and currently there are 21 weeds exhibiting resistance to glyphosate in Australia, even though the area using GM HT (tolerant to glyphosate) crops in the country is relatively small (about 0.85 million ha in 2020). In Argentina, Brazil and Canada, where GM HT crops have been widely grown for many years, the number of weed species recorded as exhibiting resistance to glyphosate in this dataset are respectively 17, 11 and 8. Some glyphosate-resistant species, such as marestail (*Conyza canadensis*), waterhemp (*Amaranthus tuberculatus*) and palmer pigweed (*Amaranthus palmeri*) in the USA, are now widespread, with the affected area being possibly within a range of 60–80% of the total combined area annually devoted to maize, cotton, and soybeans.

This resistant weed development should, however, be placed in context. All weeds have the ability to develop resistance to all herbicides and there are hundreds of resistant weed species confirmed in the International Survey of Herbicide Resistant Weeds (I Heap, as above found at www.weedscience.org). As indicated above, herbicide-resistant weeds pre-date the widespread use of GM HT crops by decades and that there are, for example, 170 weed species that are resistant to ALS herbicides (eg, imazethapyr and cloransulam methyl) and 87 weed species resistant to photosystem II inhibitor herbicides (eg, atrazine).

Where farmers using GM HT crop technology have been, and are, faced with weeds resistant to glyphosate, they are advised to be proactive and include other herbicides (with different and complementary modes of action) in combination with glyphosate and in some cases to adopt cultural practices such as plowing in their integrated weed management systems.^31,32^ This change in weed management practices have been evident from the changes in herbicide usage patterns discussed above and reflect the broader agenda of developing more integrated weed control strategies across all forms of cropping systems (not just GM HT) to minimize and slowdown the potential for weeds developing resistance to whatever form of weed control is practiced. In addition, as referred to earlier, GM HT crops tolerant to other herbicides (often stacked with glyphosate) have become available from 2016 in some countries. At the macro level, these changes have influenced the mix, total amount, cost, and overall profile of herbicides applied to GM HT crops in the last 15 years. For example, in 2020 approximately 90% of the GM HT soybean crop area in the USA was planted to varieties that were tolerant to other herbicides (in addition to tolerance to glyphosate) and even when crops were planted that were tolerant to only one herbicide, all of these crops received an additional herbicide treatment with other active ingredients (notably sulfentrazone, S metolachlor, 2,4-D, metribuzin, metsulfuron, and pyroxasulfone). This compares with only 14% of the USA GM HT soybean crop (almost all tolerant to glyphosate only) receiving a treatment of one of the next four most used herbicide active ingredients (after glyphosate) in 2006. As a result, the average amount of herbicide active ingredient applied to the GM HT soybean crop in the USA (per hectare) doubled during this period. It is also interesting to note that in the last year when glyphosate only tolerant GM HT crops dominated the USA soybean crop (2016), glyphosate accounted for a lower share of total active ingredient use on the GM HT crop (63%) than in 1998 when it accounted for 82% of total active ingredient use. This illustrates that farmers have continued to use glyphosate because of its broad-spectrum activity in addition to using other herbicides in line with integrated weed management advice. This continues in 2020, with the availability of additional options for weed control via varieties with GM HT tolerance to other herbicides. Almost all of the new GM HT seed technology used is tolerant to glyphosate and other herbicides rather than being tolerant only to other herbicides.

On the small conventional soybean crop in the USA, the average amount of herbicide active ingredient applied also doubled over the period 2006–2020. This increase in usage largely reflected a shift in herbicides used rather than increased dose rates for some herbicides. The increase in the use of herbicides on the conventional soybean crop in the USA can also be mostly associated with the on-going development of weed resistance to non-glyphosate herbicides widely used in conventional crops and highlights that the development of weed resistance to herbicides is a problem faced by all farmers, regardless of production method.

Relative to the conventional alternative, the environmental profile of GM HT crop use has, nevertheless, continued to offer important advantages and in most cases, provides an improved environmental profile compared to the conventional alternative (as measured by the EIQ indicator).

### GM IR Crops

The main way in which GM IR seed technology has impacted on the environment has been through reduced insecticide use, with the seed technology effectively replacing insecticides targeted at controlling important crop pests.

**Figure 5.**
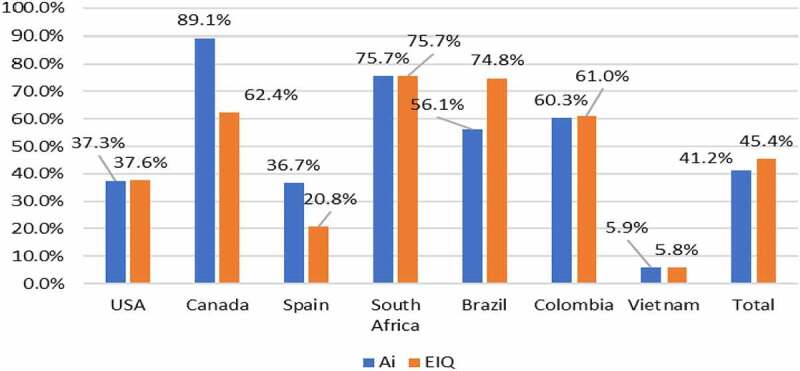
Aggregate insecticide active ingredient usage (reductions) and EIQ changes (improvements) with use of GM IR maize: by adopting country 1996–2020.

Note: see appendix 1 for details of how these changes are calculated relative to the conventional (non-GM) baseline

In maize, the use of GM IR technology since 1996, when it was first used in some countries (notably the USA), has resulted in an 85.4 million kg reduction in amount of insecticide active ingredient used (Table 4) that targets the pests that the GM IR technology control. This represents a net aggregate reduction of 41%. In terms of the environmental impact, as measured by the EIQ indicator, the use of this seed technology has delivered an improvement of 45% (Fig. 5). As the figure shows, the reduction in the amount of aggregate insecticide used on maize crops varies by adopting country (Fig. 5) and this largely reflects the extent to which the pests targeted by the GM IR technology are commonly present in crops and the extent to which maize growers had previously used insecticides to control them. For example, in the USA, no more than 10% of the maize crop typically received insecticide treatments targeted at stalk boring pests and about 30%–40% of the crop annually received treatments for rootworm pests, the main pest species controlled by GM IR seed technology. In Canada, the proportion of the crop previously treated with insecticides for the control of the main pests of the crop (stalk borers) was only 5%, whilst in Brazil about 50% of the maize crop was typically treated with insecticides for the control of stalk boring pests.

**Table 4.** GM IR maize: summary of active ingredient usage changes 1996–2020.

Country	Change in active ingredient use (million kg)
USA	−60.9
Canada	−0.91
Spain	−0.75
South Africa	−2.6
Brazil	−19.9
Colombia	−0.28
Vietnam	−0.09
**Aggregate impact: all countries**	**−85.4**

Negative sign = reduction in usage, positive sign = increase in usage Other countries using GM IR maize – Argentina, Uruguay, Paraguay, Honduras, and the Philippines, not included due to lack of data and/or little or no history of using insecticides to control these pests Changes relate to insecticides typically used to target stalk boring (lepidopteran) pests and rootworm in the USA and Canada. Some of these active ingredients are, however, sometimes used to control to other pests that the GM IR technology does not target The analysis aims to avoid over estimation of the insecticide reductions attributable to the technology by restricting the estimates to a crop base area that is equal to the smallest of the GM IR area or the maximum area of the conventional crop area (pre GM IR technology) that used to be annually treated with insecticides to control the pests that the GM IR technology aims to control. Additional detail is provided in relation to the 2020 insecticide use change estimates at the crop/country level shown in Appendix 2. Also, by focusing on this pest type-specific range of insecticides, this contributes to reducing the scope for attributing reductions in insecticide use on the crop that have occurred due to regulatory reasons (withdrawal of active ingredients)

In relation to cotton, the reduction in the volume of insecticide used with GM IR technology has been significantly larger than in the maize (Table 5). Since 1996, the technology has been responsible for a 339 million kg reduction in use of insecticide active ingredient on cotton crops around the world, equal to about a 30% reduction in total insecticide usage (by volume). This largely reflects the pest problems faced by cotton growers, with most crops suffering damage from the various bollworm/budworm pests each year. As a result, before the availability of GM IR technology, cotton crops in some countries (eg, China and Australia) were routinely sprayed 15–20 times per year in order to control these pests. With availability of GM IR seed technology the number and frequency of insecticide applications has fallen significantly to, typically less than five, and focused on control of pests that the GM IR technology does not control (eg, sucking pests). In terms of the associated environmental impact, as measured by the EIQ indicator, the use of GM IR technology in cotton has resulted in a 34% improvement (Table 5).

**Table 5.** GM IR cotton: summary of active ingredient usage and associated EIQ changes 1996–2020.

Country	Change in active ingredient use (million kg)	Change in amount of active ingredient used (%)	Change in EIQ indicator (%)
US	−8.9	−7.7	−9.8
China	−139.0	−30.1	−30.2
Australia	−14.1	−30.2	−32.1
India	−165.0	−36.4	−46.1
Mexico	−3.2	−16.6	−16.5
Argentina	−1.8	−21.9	−31.1
Brazil	−2.5	−18.4	−25.5
Colombia	−0.2	−59.3	−63.0
Burkina Faso	4.4	−28.4	−32.4
**Aggregate impact: all countries**	**−338.9**	**−29.9**	**−34.4**

Negative sign = reduction in usage or EIQ improvement. Positive sign = increase in usage or worse EIQ value Other countries using GM IR cotton – Burkina Faso, Paraguay, Pakistan, and Myanmar not included due to lack of data Values related to all insecticides (as bollworm/budworm pests are the main category of cotton pests worldwide). Some of these active ingredients are, however, sometimes used to control to other pests that the GM IR technology does not target The analysis aims to avoid over estimation of the insecticide reductions attributable to the technology by restricting the estimates to a crop base area that is equal to the smallest of the GM IR area or the maximum area of the conventional crop area (pre GM IR technology) that used to be annually treated with insecticides to control the pests that the GM IR technology aims to control. Additional detail is provided in relation to the 2020 insecticide use change estimates at the crop/country level shown in Appendix 2. Also, by focusing on this pest type-specific range of insecticides, this contributes to reducing the scope for attributing reductions in insecticide use on the crop that have occurred due to regulatory reasons (withdrawal of active ingredients)

Lastly, GM IR soybeans, which were first available for commercial use in South America in 2013, have been responsible for a 23.9 million kg (9.8% of total soybean insecticide use) reduction in insecticide use relative to the amount reasonably expected if this crop area had been planted to conventional soybeans. The largest share of this reduction occurred in Brazil (89%), which also accounted for about 80% of the total area planted to GM IR soybeans in the 2013–2020 period. In terms of the associated environmental impact, as measured by the EIQ indicator, this has improved by 17.8% (Table 6).

**Table 6.** GM IR soybeans: summary of active ingredient usage and associated EIQ changes 2013–2020.

Country	Change in active ingredient use (million kg)	Change in amount of active ingredient used (%)	Change in EIQ indicator (%)
Brazil	21.24	16.1	−53.8
Argentina	1.67	1.8	−0.9
Paraguay	0.76	6.0	−2.3
Uruguay	0.19	3.3	−1.7
**Aggregate impact**: **all countries**	**−23.86**	**−9.8**	**−17.8**

Negative sign = reduction in usage or EIQ improvement. Positive sign = increase in usage or worse EIQ value % change in active ingredient usage and field EIQ values relates to insecticides typically used to target lepidopteran pests of soybeans. Some of these active ingredients are, however, sometimes used to control to other pests that the GM IR technology does not target The analysis aims to avoid over estimation of the insecticide reductions attributable to the technology by restricting the estimates to a crop base area that is equal to the smallest of the GM IR area or the maximum area of the conventional crop area (pre GM IR technology) that used to be annually treated with insecticides to control the pests that the GM IR technology aims to control. Additional detail is provided in relation to the 2020 insecticide use change estimates at the crop/country level shown in Appendix 2. Also, by focusing on this pest type-specific range of insecticides, this contributes to reducing the scope for attributing reductions in insecticide use on the crop that have occurred due to regulatory reasons (withdrawal of active ingredients)

### Global Impacts of All Herbicide and Insecticide Use Changes with GM Crop Use

**Figure 6.**
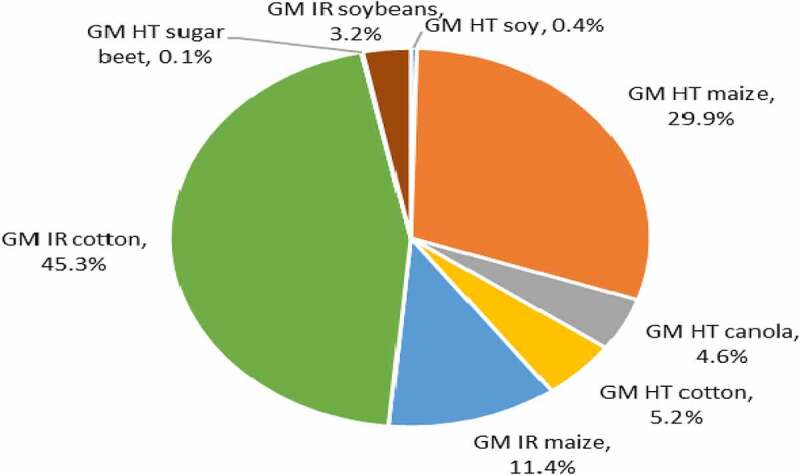
Share of aggregate active ingredient usage (reductions) by trait 1996–2020 (baseline total 748.6 million kg).

**Figure 7.**
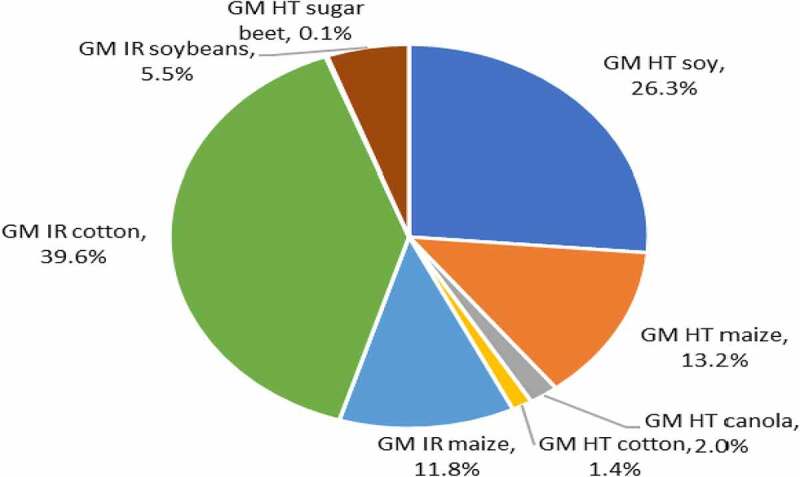
Share of aggregate EIQ changes (improvements) by trait 1996–2020.

At the global level, the analysis suggests that GM technology has contributed to a significant reduction in the negative environmental impact associated with insecticide and herbicide use on the areas devoted to GM crops. Since 1996, the use of pesticides on the GM crop area has fallen by 748.6 million kg of active ingredient (a 7.2% reduction) relative to the amount reasonably expected if this crop area had been planted to conventional crops. The largest share of this was accounted for by GM IR cotton (45%) followed by GM HT maize (30%: Fig. 6). In terms of the environmental impact associated with herbicide and insecticide use on these crops, as measured by the EIQ indicator, this improved by 17.3%, with the largest share of these improvements delivered by GM IR cotton (about 40% of the total), followed by GM HT soybeans (26%: Fig. 7).

At the country level, US farms have seen the largest environmental changes, with a 322 million kg reduction in pesticide active ingredient use (43% of the total). This is not surprising given that US farmers were first to make widespread use of GM crop technology, and for several years, the GM adoption levels in all four US crops have been in excess of 80%, and insecticide/herbicide use has, in the past been, the primary method of weed and pest control. Important environmental changes have also occurred in China and India from the adoption of GM IR cotton, with a reduction in insecticide active ingredient use of over 304 million kg (1996–2020).

Benchmarking the findings of this research with other research has been constrained by the limited nature of studies examining this issue. The findings are consistent with analysis by other authors, such as the meta (global) analysis of Klumper and Qaim, 2014^34^ or the USA-specific analysis of Fernando-Cornejo J et al., 2014.^35^ However, these studies were largely drawing on papers that examined pesticide use changes with GM crops largely from the perspective of changes in the amount of active ingredient used only (which has been the primary focus of individual crop/trait/country-level studies, where pesticide change impacts have been assessed). Where studies have explored other environmental impact indicators, the EIQ has been the main one used, not only by this author (for the reasons given in the methodology section) but by others (eg, Smyth et al., 2011,^36^ Hudson and Richards, 2014.^37^) The findings are also consistent with these studies. Where a limited number of other studies have explored the use of other indicators for assessing environmental impacts, such as Ariel and Riesgo, 2014,^38^) the findings of this research are also consistent. One study that used a different environmental indicator (Total Applied Toxicity) to examine impacts associated with the adoption of GM IR maize in the USA (Schultz et al., 2021^12^) concluded that the TAT associated with GM IR maize was no lower than that found in conventional maize and that the overall level of the TAT (for aquatic invertebrates and terrestrial pollinators) had increased between 1998 and 2016, a period during which GM IR maize’s share of the total crop increased from 19% to 79%. This study also concluded that the rise in the TAT levels was largely attributable to increasing use of neonicotinoid insecticides on the US maize crop during this period. As neonicotinoid use does not target the pests that GM IR technology targets, the increased use in this category of insecticides on the US GM IR maize crop (and on the US conventional maize crop) is unrelated to the use of GM IR maize technology (this key point undermines the authors’ claim that their findings challenge the (more widespread) claims of a decrease in the environmental impacts of insecticide use with GM crops).

## Conclusions

GM crop technology has been used by many farmers around the world for nearly 25 years and currently about 17 million farmers a year plant seeds containing this technology. The technology has helped farmers adapt their weed and pest control practices and become more efficient with their application of insecticides and herbicides. In turn, this has reduced the environmental footprint associated with the use of these crop protection products as measured in terms of the total amount of pesticide applied to the GM crop area or using the EIQ indicator.

In relation to GM HT crops, however, over reliance on the use of glyphosate by farmers, in many regions, has contributed to the development of weed resistance. As a result, farmers have, over the last 20 years, adopted more integrated weed management strategies incorporating a mix of herbicides and non-herbicide-based weed control practices. This means that the magnitude of the original environmental gains associated with changes in herbicide use with GM HT crops have diminished. In addition, the magnitude of carbon emission savings each year associated with the facilitating role of GM HT crops in the adoption of NT and RT systems is likely to have decreased as some farmers have reverted to making use of plowing as part of weed control practices of herbicide tolerant weeds. Nevertheless, despite these developments, the adoption of GM HT crop technology in 2020 continues to deliver a net environmental gain and, together with GM IR technology, continues to provide substantial net environmental benefits.

As indicated above, these findings are also consistent with analysis by other authors, though the author acknowledges that in an ‘ideal’ world, it would be possible to assess the environmental impact associated with pesticide use change with GM crop technology using a more comprehensive and transferable indicator than the one used in this study.

## APPENDIX 1: EXAMPLES OF EIQ CALCULATIONS

**Table:** Typical insecticide regimes for cotton in India 2020.

Active ingredient	Amount (kg/ha of crop)	Field EIQ/ha
*Conventional cotton*		
*Option 1*		
Imidacloprid	0.06	2.2
Thiomethoxam	0.05	1.67
Acetamiprid	0.05	1.45
Diafenthiuron	0.1	2.53
Buprofezin	0.07	2.55
Profenfos	0.81	48.28
Acephate	0.63	15.79
Cypermethrin	0.1	3.64
Metaflumizone	0.03	0.82
Novaluron	0.02	0.29
**Total**	**1.92**	**79.22**
*Option 2*		
Imidacloprid	0.06	2.2
Thiomethoxam	0.05	1.67
Acetamiprid	0.05	1.45
Novaluron	0.02	0.29
Chloripyrifos	0.39	10.58
Profenfos	0.81	48.28
Metaflumizone	0.03	0.82
Emamectin	0.01	0.29
**Total**	**1.42**	**65.58**
**Average conventional**	**1.67**	**72.40**
**Weighted average conventional**	**1.72**	**73.76**
*GM IR cotton*		
Imidacloprid	0.06	2.2
Thiomethoxam	0.05	1.67
Acetamiprid	0.05	1.45
Novaluron	0.02	0.29
Buprofezin	0.07	2.55
Acephate	0.63	15.79
**Total**	**0.89**	**23.95**
*Option 2*		
Imidacloprid	0.06	1.54
Thiomethoxam	0.05	1.67
Acetamiprid	0.05	2.30
Novaluron	0.02	0.29
**Total**	**0.18**	**5.61**
**Average GM IR cotton**	**0.53**	**14.78**
**Weighted average conventional**	**0.604**	**16.61**
**Difference GM versus conventional**	**1.12**	**57.15**

Source: Bayer India, AMIS Global/Kynetec

Note weighted average for GM IR cotton based on insecticide usage – option 1 60%, option 2 40%

**Table:** Calculation of aggregate ai and EIQ savings for 2020: example based on above example of cotton insecticides used in India 2020.

Total crop area (million ha) subject to insecticide treatment (if all crop using non-GM seed)	13
Baseline aggregate ai use (million kgs) if all conventional (basis 1.72 kg/ha)	22.4
Baseline aggregate EIQ units (million) if all conventional (basis 73.76.ha)	959.9
GM IR crop area (million ha) on which change in insecticide use applicable	12.22
Aggregate insecticide ai saving (million kg) based on average saving per ha of GM IR cotton relative to conventional cotton (1.72–0.604 = saving of 1.12 kg/ha)	13.665 (−61%)
Aggregate insecticide EIQ saving (million units) based on average saving per ha of GM IR cotton relative to conventional cotton (73.76–16.61 = saving of 57.15 EIQ units/ha)	698.4 (−72.8%)

**Table:** Estimated typical herbicide regimes for GM HT reduced/no till and conventional reduced/no till soybean production systems that will provide an equal level of weed control to the GM HT system in Argentina 2020.

	Active ingredient (kg/ha)	Field EIQ/ha value
*GM HT soybean*	3.59	54.54
Source: Kleffmann dataset on pesticide use 2018/19		
*Conventional soybean*		
*Option 1*		
Glyphosate	2.27	34.80
Metsulfuron	0.03	0.50
2,4-D	0.4	8.28
Imazethapyr	0.10	1.96
Diflufenican	0.03	0.29
Clethodim	0.19	3.23
**Total**	**3.02**	**49.06**
*Option 2*		
Glyphosate	2.27	34.80
Dicamba	0.12	3.04
Acetochlor	1.35	26.87
Haloxifop	0.18	4.00
Sulfentrazone	0.19	2.23
**Total**	**4.11**	**70.92**
*Option 3*		
Glyphosate	2.27	34.80
Atrazine	1.07	24.50
Bentazon	0.60	11.22
2,4-D ester	0.4	6.12
Imazaquin	0.024	0.37
**Total**	**4.36**	**77.01**
*Option 4*		
Glyphosate	2.27	34.80
2,4-D amine	0.4	8.28
Flumetsulam	0.06	0.94
Fomesafen	0.25	6.13
Chlorimuron	0.05	0.96
Fluazifop	0.12	3.44
**Total**	**3.15**	**54.54**
*Option 5*		
Glyphosate	2.27	34.80
Metsulfuron	0.03	0.50
2,4-D amine	0.8	16.56
Imazethapyr	0.1	1.96
Haloxifop	0.18	4.00
**Total**	**3.38**	**57.82**
*Option 6*		
Glyphosate	2.27	34.80
Metsulfuron	0.03	0.50
2,4-D amine	0.8	16.56
Imazethapyr	0.1	1.96
Clethodim	0.24	4.08
**Total**	**3.44**	**57.90**
**Average all six conventional options**	**3.577**	**61.21**
**Weighted average**	**3.616**	**62.04**

Sources: AAPRESID, Kleffmann/Kynetec, Bayer Argentina

Weights (based on expected usage): option 1 10%, options 2 to 5 17.5% each, option 6 20%

GM HT average use based on data from about 20 herbicides, of which the most used 10 active ingredients account for 99% of total usage by weight of ai

**Table:** Calculation of aggregate ai and EIQ savings for 2020: example based on above example of soybean herbicides in Argentina 2020.

Total crop area (million ha) subject to herbicide treatment (if all crop using non-GM seed)	16.47
Baseline aggregate ai use (million kgs) if all conventional (basis 3.616 kg/ha)	59.54
Baseline aggregate EIQ units (million) if all conventional (basis 62.04 ha)	1,021.5
GM IR crop area (million ha) on which change in herbicide use applicable	15.99
Aggregate herbicide ai saving (million kg) based on average saving per ha of GM HT cotton relative to conventional soybeans (3.616–3.59 = saving of 0.026 kg/ha)	0.434 (−0.7%)
Aggregate herbicide EIQ saving (million units) based on average saving per ha of GM HT soybeans relative to conventional soybeans (62.04–54.53 = saving of 7.51 EIQ units/ha)	120.04 (−11.8%)

**Table:** National-level changes in herbicide ai use and field EIQ values for GM HT soybeans in Canada 1997–2020.

Year	ai saving (kg)	EIQ saving (units)	% decrease in ai (- = increase)	% EIQ saving
1997	530	20,408	0.03	0.06
1998	25,973	1,000,070	1.8	3.0
1999	106,424	4,097,826	7.4	11.9
2000	112,434	4,329,247	7.4	11.9
2001	169,955	6,544,073	11.1	17.9
2002	230,611	8,879,609	15.7	25.4
2003	276,740	10,655,776	18.5	29.8
2004	351,170	13,521,703	20.4	32.8
2005	373,968	14,399,532	22.2	35.8
2006	84,130	10,190,844	4.8	24.5
2007	75,860	9,167,256	4.5	22.7
2008	96,800	11,725,560	5.6	28.5
2009	103,374	12,521,832	5.2	26.5
2010	113,729	13,776,201	5.4	27.3
2011	97,749	11,840,550	4.4	22.2
2012	119,977	14,533,032	5.0	25.3
2013	133,634	16,187,269	5.0	25.3
2014	149,969	18,165,957	3.7	24.1
2015	65,157	12,734,412	1.7	16.7
2016	67,142	13,122,473	1.7	17.1
2017	84,235	16,463,031	1.6	16.1
2018	73,787	14,421,142	1.7	16.3
2019	69,947	13,670,602	1.8	17.3
2020	61,434	12,006,856	1.7	16.9

Sources: Own calculations based on data from George Morris Center 2004,^39^ Weed Control Guide Ontario (updated annually), extension and industry advisors

**Table:** National-level changes in herbicide ai use and field EIQ values for GM HT soybeans in Argentina 1996–2020.

Year	ai saving (kg negative sign denotes increase in ai use)	EIQ saving (units)	% decrease in ai (- = increase)	% EIQ saving
1996	9,250	369,260	0.0	0.1
1997	439,000	17,524,880	2.1	4.9
1998	1,200,000	47,904,000	5.4	12.4
1999	1,660,000	66,267,200	6.3	14.6
2000	2,250,000	89,820,000	6.6	15.3
2001	2,731,250	109,031,500	7.4	17.1
2002	3,111,500	124,211,080	7.5	17.3
2003	3,307,500	132,035,400	7.6	17.6
2004	3,514,500	140,298,840	7.6	17.6
2005	3,800,000	151,696,000	7.8	18.0
2006	3,960,000	158,083,200	7.6	17.6
2007	−2,462,929	37,108,129	−4.6	4.0
2008	−2,489,981	37,515,712	−4.6	4.0
2009	−2,727,300	41,091,320	−4.6	4.0
2010	−2,044,167	121,864,264	−3.5	12.1
2011	−4,419,360	18,414,000	−7.4	1.8
2012	−4,620,780	19,253,250	−7.4	1.8
2013	−5,754,541	9,933,052	−10.3	1.0
2014	−5,763,282	9,948,140	−10.3	1.0
2015	−5,652,562	9,757,023	−10.3	1.0
2016	501,668	138,799,176	0.7	12.0
2017	442,334	122,383,135	0.7	12.1
2018	448,874	124,192,360	0.8	12.1
2019	452,815	125,282,774	0.7	12.1
2020	433,853	120,036,613	0.8	11.8

Sources: own calculations based on data from AMIS Global & Kleffmann (private market research data on crop pesticide use), Qaim and T raxler 2005,^22^ Rodriguez A et al (2021)^40^ plus Monsanto (and Bayer) Argentina (personal communications 2007, 2009, 2011, 2013, 2014, 2015, 2016, 2018, 2020)

## APPENDIX 2: SUMMARY OF 2020 CALCULATIONS OF ENVIRONMENTAL IMPACT ASSOCIATED WITH PESTICIDE USE CHANGES

**Table:** GM IR maize 2020.

Country	Area of trait (‘000 ha)	Maximum area treated for stalk boring pests: pre-GM IR (‘000 ha)	Average ai use GM crop (kg/ha)	Average ai use if conventional (kg/ha)	Average field EIQ/ha GM crop	Average field EIQ/ha if conventional	Aggregate change in ai use (‘000 kg)	Aggregate change in field EIQ/ha units (millions)
US	27,367	3,337	0.36	0.965	11.4	41.33	−3,304	−164.8
Canada	1,136	70	0.04	0.64	4.8	24.8	−41	−1.1
Argentina	5,952	0	0	0	0	0	0	0
Philippines	683	Very low – assumed zero	0	0	0	0	0	0
South Africa	2,068	1,528	0	0.08	0	3.8	−165	−6.0
Spain	98	35	0.37	1.33	0.9	26.9	−33.2	−0.9
Uruguay	115	Assumed to be zero: as Argentina	0	0	0	0	0	0
Brazil	18,045	9,518	0 targeted at stalk boring pests	0.21 targeted at stalk boring pests	0 targeting stalk boring pests	17.18	−2,033	−163.5
Colombia	96	57	0.07 targeted at stalk boring pests	0.281 targeted at stalk boring pests	1.9 targeting stalk boring pests	9.25	−12	−0.4
Vietnam	92	725	0.072	0.36	2.84	4.06	26.4	−1.0

Notes: Other countries: Honduras, Paraguay and EU countries: not examined due to lack of data (Honduras and Paraguay) or very small area planted (EU countries other than Spain)

Baseline amount of insecticide active ingredient shown in Canada refers only to insecticides used primarily to control stalk boring pests

**Table:** GM IR cotton 2020.

Country	Area of trait (‘000 ha)	Average ai use GM crop (kg/ha)	Average ai use if conventional (kg/ha)	Average field EIQ/ha GM crop	Average field EIQ/ha if conventional	Aggregate change in ai use (‘000 kg)	Aggregate change in field EIQ/ha units (millions)
US	3,030	0.83	0.97	88.97	94.97	−424	−18.2
China	3,087	1.57	2.74	73.02	103.41	−3,612	−93.8
Australia	267	0.91	2.01	42.13	104.1	−435	−16.5
Mexico	103	3.60	5.22	127.2	183.8	−168	−5.8
Argentina	441	0.41	0.736	15.12	38.16	−144	−10.2
India	12,220	0.604	1.72	16.61	73.76	−13,665	−958.9
Brazil	1,169	0.41	0.736	15.1	38.20	−382	−26.9
Colombia	8	0.35	0.69	8.49	20.29	−1.6	−35.2

Notes: Due to the widespread and regular nature of bollworm and budworm pest problems in cotton crops, GM IR areas planted are assumed to be equal to the area traditionally receiving some form of conventional insecticide treatment

South Africa, Pakistan, and Myanmar not included in analysis due to lack of data on insecticide use changes. Burkina Faso not included as not GM IR crop grown in 2020

Brazil: due to a lack of data, usage patterns from Argentina have been assumed

**Table:** GM HT soybean 2020.

Country	Area of trait (‘000 ha)	Average ai use GM crop (kg/ha)	Average ai use if conventional (kg/ha)	Average field EIQ/ha GM crop	Average field EIQ/ha if conventional	Aggregate change in ai use (‘000 kg)	Aggregate change in field EIQ/ha units (millions)
US	33,315	2.83	2.78	51.59	54.66	+1,566	−96.1
Canada	1,755	1.72	1.755	28.02	34.86	−61.4	−12.0
Argentina	15,989	3.59	3.616	54.54	62.04	+434	−120.0
Brazil	36,665	3.10	3.16	48.97	54.72	−2,115	−210.7
Paraguay	3,118	3.57	3.3	44.43	51.84	+845	−23.1
South Africa	786	1.68	1.95	28.73	42.51	−210	−10.8
Uruguay	1,067	3.01	3.04	46.23	62.04	+29	−16.9
Bolivia	1,348	3.57	3.3	44.43	51.84	+365	−10.0

Notes: Due to lack of country-specific data, usage patterns in Paraguay assumed for Bolivia. Industry sources confirm this assumption reasonably reflects typical usage. Mexico did not plant any GM soybeans in 2020.

**Table:** GM IR (Intacta) soybeans 2020.

Country	Area of trait (‘000 ha)	Average ai use GM crop (kg/ha)	Average ai use if conventional (kg/ha)	Average field EIQ/ha GM crop	Average field EIQ/ha if conventional	Aggregate change in ai use (‘000 kg)	Aggregate change in field EIQ/ha units (millions)
Brazil	23,680	1.43	1.6	30.65	47.90	−4,084	−408.5
Paraguay	1,332	0.23	0.31	6.77	9.88	−106	−4.1
Argentina	4,117	0.23	0.31	6.77	9.88	−329	−12.8
Uruguay	352	0.23	0.31	6.77	9.88	−28	−1.2

**Table:** GM HT maize 2020.

Country	Area of trait (‘000 ha)	Average ai use GM crop (kg/ha)	Average ai use if conventional (kg/ha)	Average field EIQ/ha GM crop	Average field EIQ/ha if conventional	Aggregate change in ai use (‘000 kg)	Aggregate change in field EIQ/ha units (millions)
US	29,703	3.47	3.74	67.09	75.72	−7,962	−256.4
Canada glyphosate tolerant	1,346	2.81	2.97	37.10	67.91	−213	−18.15
Canada glufosinate tolerant	21	1.97	2.97	43.38	67.91	−27	−0.7
Argentina	6,272	3.99	3.53	71.8	73.61	+2,909	−11.3
South Africa	2,090	2.33	2.22	39.46	46.45	+230	−14.6
Brazil	16,459	2.81	2.81	48.86	56.45	No change	−124.9
Uruguay	126	3.99	3.53	71.8	73.61	+58	−0.2
Philippines	686	1.92	1.90	32.93	43.41	+10	−7.2
Vietnam	92	2.08	2.88	37.94	59.74	−73	−2.0
Colombia	109	2.07	2.51	40.98	59.05	−49	−2.0

Notes: Uruguay – based on Argentine data – industry sources confirm herbicide use in Uruguay is very similar

**Table:** GM HT cotton 2020.

Country	Area of trait (‘000 ha)	Average ai use GM crop (kg/ha)	Average ai use if conventional (kg/ha)	Average field EIQ/ha GM crop	Average field EIQ/ha if conventional	Aggregate change in ai use (‘000 kg)	Aggregate change in field EIQ/ha units (millions)
US	3,133	4.62	4.5	86.55	90.28	+376	−11.7
S Africa	16	1.80	1.81	27.59	31.86	−0.2	−0.1
Australia	280	5.26	7.47	90.22	143.43	−617	−14.9
Argentina	450	4.06	4.72	63.96	78.40	−296	−6.5
Colombia	109	1.79	2.30	28.03	38.21	−55	−1.1

Notes: Mexico not included due to lack of data on herbicide use applicable for conventional cotton

**Table:** GM HT canola 2020.

Country	Area of trait (‘000 ha)	Average ai use GM crop (kg/ha)	Average ai use if conventional (kg/ha)	Average field EIQ/ha GM crop	Average field EIQ/ha if conventional	Aggregate change in ai use (‘000 kg)	Aggregate change in field EIQ/ha units (millions)
US glyphosate tolerant	162	0.98	1.06	15.04	22.34	−13	−1.2
US glufosinate tolerant	527	0.27	1.06	10.64	22.34	−416	−6.2
Canada glyphosate tolerant	3,203	0.916	0.84	14.02	14.52	−256	−1.6
Canada glufosinate tolerant	4,784	0.55	0.84	11.25	14.52	−1,650	−15.7
Australia glyphosate tolerant	562	0.94	1.46	15.03	29.62	−295	−8.2

**Table:** GM herbicide tolerant sugar beet 2020.

Country	Area of trait (‘000 ha)	Average ai use GM crop (kg/ha)	Average ai use if conventional (kg/ha)	Average field EIQ/HA GM crop	Average field EIQ/ha if conventional	Aggregate change in ai use (‘000 kg)	Aggregate change in field EIQ/ha units
US	462	3.57	3.42	62.63	62.52	+69	−0.05

## Appendix 3.

**Table:** DATA SOURCES FOR PESTICIDE USE CHANGE ESTIMATES.

	Sources of data for assumptions
US	Gianessi & Carpenter 1999^41^ Sankala & Blumenthal (2003^26^ & 2006^27^) Johnson S & Strom S 2007^28^ Own analysis (2010–2020) All of the above mainly for conventional regimes (based on surveys and consultations of extension advisors and industry experts) Kynetec – private market research data on pesticide usage. Is the most comprehensive dataset on crop pesticide usage at the farm level and allows for disaggregation to cover biotech versus conventional crops. This source primarily used for usage on GM traits
Argentina	AMIS Global & Kleffmann - private market research data on pesticide use. Is the most detailed dataset on crop pesticide use AAPRESID (no till farmers association) – personal communications 2007 Monsanto Argentina (personal communications 2005, 2007, 2009, 2010, 2011, 2012, 2013, 2014, 2015, 2016, 2017) Qaim M & De Janvry A 2005^42^ Qaim M & Traxler G 2005^22^
Brazil	AMIS Global & Kleffmann - private market research data on crop pesticide use. Is the most detailed dataset on crop pesticide use Monsanto Brazil 2008^43^ Galveo A,(2009^44^and 2012^45^) plus personal communications Monsanto Brazil (personal communications 2007, 2009, 2011, 2013, 2014, 2015, 2016)
Uruguay	Kleffmann and as Argentina for conventional
Paraguay	As Argentina for conventional soybeans (over the top usage), Kleffmann for GM HT soybean
Bolivia	As Paraguay: no country-specific data identified
Canada	George Morris Center 2004^39^ Canola Council 2001^46^ Smyth S et al 2011^36^ Weed Control Guide Ontario (updated annually)
S Africa	Monsanto S Africa (personal communications 2005, 2007, 2009, 2010, 2011, 2012, 2014, 2015, 2016) Ismael Y et al 2002^47^ Kleffmann
Romania	Kleffmann, Brookes 2005^18^
Australia	Kleffmann Doyle et al (200 3^48^ and 2005^49^) CSIRO 2005^50^ Monsanto Australia (personal communications 2005, 2007, 2009, 2010, 2011,2012, 2014, 2016, 2017) Fisher J & Tozer P 2009^51^
Spain	Brookes (2008^24^ and 2019^52^)
China	Kleffmann Pray et al 2002^23^ Monsanto China personal communication (2007, 2009, 2010, 2011, 2013, 2014, 2016, 2017)
Mexico	Monsanto Mexico 2005^53,^2007^54,^2008^55,^2009^56,^2012^57,^2013^58,^2015^59,^2016^60,^2017^61^ Traxler G et al ^200162^
India	Kleffmann, Kynetec APCOAB 2006^63^ IMRB 2006^64,^2007^65^ Monsanto India (2007, 2008, 2009, 2010, 2011, 2013, 2016, 2017) – personal communications
Vietnam	Kynetec, Brookes 2017^66,67^
Philippines	Kynetec, Monsanto Philippines personal communication and survey of GM HT growers (2017 unpublished)
Colombia	Kynetec, Brookes 2020^68^
